# Supplementary material for: Natural Occurrence of Beauvericin and Enniatins in Corn- and Wheat-Based Samples Harvested in 2017 Collected from Shandong Province, China
Source: Toxins (Basel). 2018 Dec 27;11(1):9. doi: 10.3390/toxins11010009 (PMC6356366; doi:10.3390/toxins11010009)
Supplement: Supplementary file 1 [file toxins-11-00009-s001.pdf]

Supplementary Materials: Running Title: BEA and ENNs in 2017 Chinese Corn- and Wheat-Based Samples

Natural Occurrence of Beauvericin and Enniatins in Corn- and Wheat-Based Samples Harvested in 2017 Collected from Shandong Province, China

Xiaomin Han, Wenjing Xu, Jing Zhang, Jin Xu and Fengqin Li

Table S1. Important MRM parameters of MS/MS conditions for BEA and ENNs.

**Mycotoxin**	**Parent Ion Form**	**Parent Ion(m/z)**	**Daughter ion(m/z)**	**DP [f] (V)**	**CE [g] (V)**	**CXP [h] (V)**	**EP [i](V)**	**Ion Source**
BEA [a]	$[M+H]^+$	801.4	244.1*/262.1	100	40/40	12/12	**7/7**	ESI^+ [j]
ENA [b]	$[M+H]^+$	699.5	210.1*/228.2	100	40/40	10/12	**7/7**	ESI^+
ENA_1 [c]	$[M+H]^+$	685.5	210.2*/228.2	84	38/38	7/7	**7/7**	ESI^+
ENB [d]	$[M+H]^+$	657.5	196.2*/214.1	90	38/38	9/10	**7/7**	ESI^+
ENB_1 [e]	$[M+H]^+$	671.5	196.2*/210.2	88	40/40	8/8	**7/7**	ESI^+

[a]: BEA = beauvericin; [b]: ENA = enniatin A; [c]: ENA_1 = enniatin A_1; [d]: ENB = enniatin B; [e]: ENB_1 = enniatin B_1; [f]: DP = declustering potential; [g]:CE = collision energy; [h]: CXP = collision cell exit potential; [i]: EP = entrance potential; [j]: ESI^+ = electrospray ionization in positive mode.
